# Effect of Processed Beverage By-Product-Based Diets on Biological Parameters, Conversion Efficiency and Body Composition of *Hermetia illucens* (L) (Diptera: Stratiomyidae)

**DOI:** 10.3390/insects12050475

**Published:** 2021-05-20

**Authors:** Vassilios Sideris, Maria Georgiadou, Georgios Papadoulis, Konstantinos Mountzouris, Antonios Tsagkarakis

**Affiliations:** 1 Laboratory of Agricultural Zoology and Entomology, Agricultural University of Athens, 118 55 Athens, Greece; vsideris@aua.gr (V.S.); gpapadoulis@aua.gr (G.P.); 2 Laboratory of Nutritional Physiology & Feeding, Agricultural University of Athens, 118 55 Athens, Greece; mgeo@aua.gr (M.G.); kmountzouris@aua.gr (K.M.)

**Keywords:** black soldier fly, upcycling, spent coffee grounds, brewer’s spent grains, protein conversion, bioconversion rate, substrate mass reduction

## Abstract

**Simple Summary:**

The Black Soldier Fly insect (BSF) *Hermetia illucens* (L.) (Diptera: Stratiomyidae) metabolizes low degraded ingredients and converts them into larval enriched nutrients. In the present study, the effect of processed beverage by-products, such as spent coffee grounds and brewer’s spent grains and their mixtures on biological parameters (larval development, survival), conversion efficiency and body composition of the BSF insect was evaluated. The effect of different rearing densities of BSF on the above parameters were also studied. Insect larvae were reared successfully in all tested diets, except for sole spent coffee grounds. Substrate mass reduction, protein conversion and bioconversion rates were higher on the reference feed, followed by brewer’s spent grains and brewer’s spent grains—spent coffee grounds mixture enriched with brewer’s yeast. Density did not affect any larval parameter except for fat. Our results illustrate that low value beverage by-products can be successfully utilized as constituents of a successful BSF diet.

**Abstract:**

The effect of spent coffee grounds (SCG), brewer’s spent grains (BSG) and their mixtures with the addition of brewer’s yeast (BY) were tested in two rearing densities of the Black Soldier Fly, *Hermetia illucens* (L.). Different treatments were investigated on larval development, survival, yield, protein conversion (PrCR) and bioconversion rate (BCR), substrate mass reduction and body composition of the insect. BSF larvae were able to develop sufficiently in all diets, except on sole SCG. The addition of BY enhanced the performance properties of diets, especially in the case of SCG, where larvae underperformed. Substrate mass reduction, PrCR and BCR were affected only by feed and exhibited higher values on reference feed, followed by BSG and SCG+BSG enriched with BY. Density did not have a significant effect on various larval nutrients, except for fat, which was higher on larvae fed enriched feeds with BY and in the 300 larval density. The interaction between feed and density strongly affected the nitrogen and protein levels, larval yield and ash. Generally, diets which contained SCG exhibited high larval crude protein levels. Our results illustrate that low value beverage by-products can be successfully utilized as constituents of a successful BSF diet.

## 1. Introduction

“Upcycling” is a part of our global contemporary philosophy and a major trend in sustainable waste management. The essence of this procedure is the transformation of qualitatively low value materials into something of higher merit [1]. New methods of waste management are being explored as humanity is struggling with the increasing population and its welfare maintenance [2]. These challenges should be addressed both on a sustainable food production basis—with facilitation of alternative sources of protein—and on the management of food waste basis. Furthermore, additional challenges such as mitigation of climate crisis and preservation of natural resources should be taken into account [3,4,5].

Estimates of European food waste levels for 2012 were approximately 88 million tons from which 53% originated from households and 19% from the food and beverage processing industry, which are the highest waste contributing sectors [6]. The most abundant by-products generated in the latter class are brewer’s spent grains (BSG) followed by brewer’s yeast which are residues produced by malted grain during the brewing process and spent coffee grounds (SCG) [7,8]. According to the international coffee organization, the global coffee consumption increased from 2014 to 2018 by 2.1%, reaching about 161 billion bags (60 kg per bag) the last year [9], while the respective annual average beer production is estimated to be roughly 1.97 billion hl. Approximately, 20 Kg of wet BSG are being produced per 100 Lt of brewed beer and to date, Europe contributes to an annual amount of ~3.4 million tons [10].

Up to date, due to economic and technological constrains, the main uses of the aforementioned by-products has been limited to direct animal feeding (especially to ruminants and fish aquaculture), fuel and biodiesel production, composting or even disposal as waste in landfills [11,12]. The chemical analysis has shown high percentages of non-starch polysaccharides and lignin, while mediocre levels of crude protein, fluctuating for BSG from 19%–30% and SCG between 12.8%–16.9% [7,10]. Considering the above, “Upcycling” of low value heterogeneous food and beverage by-products is an appealing approach towards sustainable production and waste management.

The adephagous insect *Hermetia illucens* (L.) (Diptera: Stratiomyidae), commonly known as black soldier fly (BSF), is an excellent aspirant for the utilization of low degraded ingredients such as animal manure, kitchen wastes, fruits and vegetables, as well as animal materials and their conversion into larval enriched nutrients [13,14,15,16]. Recent studies have indicated the successful exploitation of coffee by-products of an affluent source of insect diet [17,18]. A number of advantages of *H. illucens* make this insect ideal for this purpose. Adult flies, having no functional mouthparts, do not feed, surviving on fat stores during larval stage and thus are not considered as pests, disease vectors or nuisances of any kind [19]. Furthermore, BSF larvae empty their gut during the last instar (pre-pupa) stage prior to migration [20] while it has been suggested that they accelerate the reduction of *Salmonella* spp. in the rearing substrate [19,21].

Several studies have focused on the impact of biological parameters, i.e., biotic and abiotic, of *H. illucens* on its life cycle, yield and body composition. Experiments have revealed strong influence of larval density in relation with dietary nutrient concentration and feeding rate on the BSF performance, the bioconversion efficiency, the larval protein and crude fat [22,23].

In an attempt to achieve greater feed conversion efficiencies, larval performance and exploitation of problematic fibrous vegetal by-products, we tested individually or combined nutritionally low and enriched fibrous by-products from the beverage sector. The overall purpose of the current study was to evaluate the efficiency of BSF larvae when consuming three types of beverage sector by-products, i.e., brewer’s spent grains, brewer’s yeast, spent coffee ground and their combinations, at two larval densities. The specific objectives of this experiment were to define and compare the possible interaction between feed quality and density on BSF larval performance, body composition and conversion efficiencies of the candidate diets.

## 2. Materials and Methods

Insects for the present study were obtained from a BSF stock colony established in the Laboratory of Agricultural Zoology and Entomology of the Agricultural University of Athens, Greece. Initial insects were purchased from “Illucens GmbH” (Ahaus, Germany) in March 2019.

### 2.1. Origin and Preparation of the Insect Diets

Greek beverage by-products used in BSF larvae diets included: spent coffee grounds (SCG)- arabica variety (small retail coffee shop, Athens, Greece), brewer’s spent grains (BSG) and autolyzed brewer’s yeast (BY) (Athenian Brewery S.A., Athens, Greece). Layer feed ΒΩ-321 (Viozois S.A., Athens, Greece) was used as a reference feed. All the above substrates were oven dried in a drying chamber (WTC Binder 78532, Tuttlingen, Germany), at 70 °C for 3–4 days until constant weight. After cooling at room temperature, the exact moisture content of samples was determined. Subsequently, to obtain homogeneity and size similar to SCGs, BSGs were grinded to 1 mm particle size using a mill (CT 193 Cyclotec, Foss Analytical AB, Höganäs, Sweden). All ground substrates were stored under refrigeration at −10 °C along with brewer’s yeast which was already stored in a freezer until use. The above ingredients as well as reference feed were used as a basis for seven diets that were prepared: (1) SCG, (2) BSG, (3) SCG + BSG (1:1), (4) SCG + BY, (5) BSG + BY, (6) SCG + BSG + BY (1:1) and (7) reference feed: Chicken feed, with a 70% moisture content achieved either by addition of water or brewer’s yeast. Each diet was mixed manually, weighed on a precision balance (Vector BL 1500, Jiangsu, China) and placed at a separate plastic bag corresponding to the respective feeding day and kept refrigerated until 24 h before use.

### 2.2. Experimental Procedure

The experimental procedure and calculations were based on established protocols described in detail by Bosch et al. [24]. Baseline larvae production was initiated by placing eggs, which were obtained from the maintained colony after a 6 h egg laying interval, in plastic containers with 10 g moistened chicken feed (reference feed) and transferring them in a controlled climate chamber at 30 °C, 70% R.H. and 24 D photoperiod. Incubated eggs were continuously inspected for eclosion. Emerged larvae were left to feed for 5 days prior to their introduction to the diets under evaluation.

In each treatment (diet) appropriate numbers of five-day-old larvae were hand-counted with sterile forceps and transferred to the containers in order to achieve the desired density. The experiment comprised of 14 treatments with 4 replicates, i.e., seven substrates in two densities of 200 and 300 larvae per container translating to 1.45 and 2.17 larva/cm^2^, respectively. The tested densities were selected according to information reported in previous studies on BSF [19,25]. The total amount of feed given per larva was 240 mg in DM. Feed quantities were weighed in a precision balance (Vector BL 1500) and placed inside plastic containers 14.5 × 9.5 × 5 cm with a lid which had a 5 × 3 cm window covered with a muslin fabric attached with thermic glue in order to facilitate ventilation and prevent larvae from crawling out. Larvae were added inside the containers immediately after introducing the diets. The experiment was conducted in a controlled climate chamber at 27 °C, 70% R.H. in absolute dark as the larvae have been reported to be photophobic [26]. Feeding was repeated once, 3 days later. Sampling of larvae was conducted by randomly selecting 30 larvae counted with entomological forceps before feeding and every 2nd or 3rd day after the last feed. The larvae were washed thoroughly under running lukewarm water to remove feed and fecal residues from their bodies, while their integument was dried with paper tissue and then weighed on an analytical balance (ACS 80-4N, Kern and Sohn GmbH, Balingen, Germany). Larvae after weighting were returned to the experimental container. Visual observation for pre-pupae started from sixth day and continued in 1–2 day intervals. In sole SCG treatments, 15 g of water was added in the feed every 3rd day. The experiment ended, according to the harvest criterion, when at least 5% of the larvae reached the 6th instar stage. Pre-pupae were distinguished by the characteristic dark brown color, typical for the 6th instar [27]. At the end of the experiment pre-pupae and larvae were harvested, washed, dried and weighed separately. After weighing, larvae from each treatment were placed in labeled airtight bags, inactivated at −10 °C and oven dried at 70 °C until constant weight. After harvesting, every container with the residue (unconsumed feed + excretory products) was weighed and oven dried at 70 °C. Baseline larvae and the aforementioned samples were kept under refrigeration until chemical analysis.

### 2.3. Chemical Analysis

Both larva and substrate chemical analyses were carried out at the Laboratory of Nutritional Physiology and Feeding, AUA (Athens, Greece). All samples were oven dried at 70 °C until constant weight. Total nitrogen content was determined using the Kjeldahl method [28] and converted to crude protein (CP) content by multiplication with factor 4.76 for the larvae, 5.6 for chicken feed and 5.4 for the rest of the substrates [29,30]. For the larvae, non-protein nitrogen (NPN) was determined using the trichloroacetic acid method (TCA) [31] and calculated after subtracting the protein nitrogen from total nitrogen. Protein nitrogen determined using the Kjeldahl method [26] was converted to true protein by multiplication with factor 4.76. Crude fat (CF) was analyzed according to the Soxhlet method [32]. Ash was determined by incineration [33] at 550 °C for 4 h in a combustion oven. For substrates, fibrous fractures were determined by the NDF and ADF analysis [34]. Non-fiber carbohydrates were calculated by the following equation: 100 − (CP% + CF% + NDF% + Ash%). All nutrient contents reported are on DM basis. Chemical analysis on food composition of tested diets is shown in Table 1.

### 2.4. Calculations and Statistics

In diet preparations, sampling of the substrates was conducted before they were frozen. SCG and BSG had approximately 5% of water content after drying. Diets were prepared accordingly in order to contain 70% moisture per weight.

In order to define how much water was required to be added, we used the following Equation (1):

(1) $$y=\frac{x\times moisture}{\left(x+w\right)}\;\%$$

where *x*: mass of substrate (g)

*w*: added water needed (g)

*y*: desired Dry Matter

Water content after combining two different moistened substrates was calculated according to the Equation (2):

(2) $$Moisture\;content=\frac{\left(\left(Mass1\times moisture1\right)+\left(mass2\times moisture2\right)\right)}{mass1+mass2}\;(g)$$

Equation (2) was used to determine the BY needed quantities in order to achieve 70% feed moisture.

Larval counts, larval, pre-pupal and residue dry weight were used for the calculations.

Larval survival rates were calculated using Equation (3) as the ratio of larvae at the end (larvae end) and the beginning (larvae beg) of the experiments:

(3) $$Survival\;Rate\;\%=\frac{larvae\;end}{larvae\;beg}\times 100$$

Mass reduction or overall degradation of the substrate was calculated using Equation (4) as the ratio of residue’s dry mass (residue mass) to the dry mass of the total feed (feed mass) provided:

(4) $$Mass\;Reduction\;\%=\left(1-\frac{residue\;mass\left(g\right)}{feed\;mass\left(g\right)}\right)\times 100$$

The bioconversion rate was calculated using Equation (5), in which the larval dry weight gain (weight gain) was calculated as the difference between the final larval dry weight and the initial larval dry weight multiplied by the number of larvae at the end of the experiment, subtracted with total feed mass provided in DM):

(5) $$Bioconversion\;Rate\;\%=\frac{Weight\;gain\left(g\right)}{feed\;mass\left(g\right)}\times 100$$

The protein conversion ratio was calculated using Equation (6) as the ratio of the amount of larval crude protein accumulated (protein gain) to crude protein in the feed provided (feed mass). Larval protein accumulation was calculated as the difference between the amount of final larval protein and the initial larval crude protein. The amount of feed protein was calculated by multiplying the feed protein content with the feed mass added in DM:

(6) $$PrCR\%=\frac{Protein\;gain\left(g\right)}{Protein\;in\;feed\;mass\left(g\right)}\times 100$$

The statistical analysis was performed by JMP 14.0 (SAS Institute Inc., Cary, NC, USA). The results of all experiments were analyzed by two-way analysis of variance (ANOVA) to compare the effect of feed and density as factors. Analysis followed by post hoc Tukey’s test HSD (honestly significant differences) to compare the significance (*p*) between the means of different groups. *p* < 0.05 was considered to indicate a significant difference between the values compared. Furthermore, correlation and linear regression tests were conducted in order to observe how feed ingredients affect mean larval dry mass and conversion efficiencies.

## 3. Results

### 3.1. Effect of Diet on Development Time, Survival Rate, Dry Mater, Yield (DM), Mean Wet and Dry Mass of the Larvae

**Figure 1:**
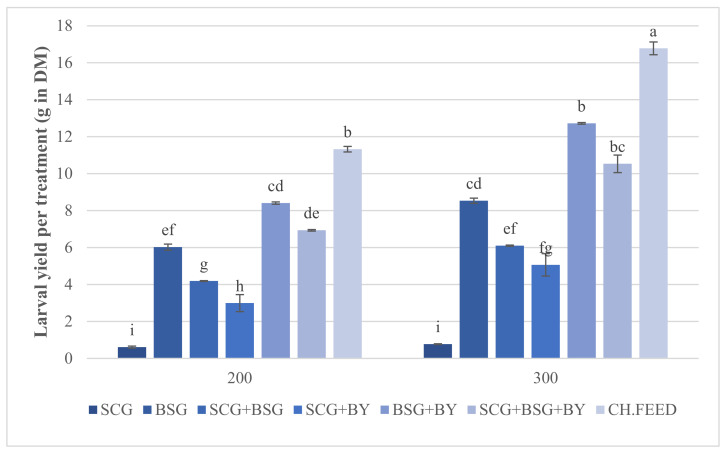
Effect of interaction between feed and density on larval yield (g in DM), (mean ± standard error, *n* = 4). Mean values followed by the same letter do not differ significantly (*p* < 0.05).

Development time needed in order for larvae fed on different diets to fulfill harvest criteria ranged between 8 and 17 days (Table 2). In the case of sole spent coffee grounds, up to the 35th day harvest criteria were not met and thus this treatment was considered incomplete. The shortest development time (8 days) was achieved by the BSG + BY diet, followed by BSG where development was completed in 9 days. Larvae exhibited the longest development period (17 days) when fed with SCG + BY. The full factorial ANOVA showed that diet had a significant effect on larval mean wet mass (F_6,42_ = 444.95, *p* < 0.0001), pre-pupal mean wet mass (F_5,24_ = 106.53, *p* < 0.0001) and larval mean dry mass (F_6,42_ = 573.77, *p* < 0.0001). A significant interaction was also found between diet (Feed) and density factors on larval dry mass (F_6,42_ = 4.55, *p* = 0.0012), survival rate (F_6,42_ = 2.49, *p* = 0.0372) and larval yield ((F_6,42_ = 2.6223, *p* = 0.0299). No significant effect of density on any of the larval performance parameters was observed. Survival rate did not differ between different diets except for SCG + BSG 300 which had a significantly lower survival rate (87.42%), compared to the rest of the diets (Table 2). Additionally, larval dry matter was significantly reduced in the cases of SCG, SCG + BSG and their combinations with BY diets at both densities, compared to the reference feed (CH.FEED) and the rest of the diets (Table 2). Larvae fed on chicken feed reached the highest larval yield (DM) and differed significantly at both densities, BSG and SCG + BSG with BY did not differ significantly compared with the same densities, while larval biomass in sole SCG at both densities remained poor, with no statistical difference (Table 2; Figure 1). The highest mean larval wet and dry masses were observed on chicken feed 0.177 g and 0.056 g, respectively, while the lowest ones on sole SCG, SCG + BSG and SCG + BY (Table 3). Concerning the mean pre-pupal wet mass, larvae reared on chicken feed grew larger with an average wet mass of 0.1601 g followed by BSG and SCG + BSG mixed with BY (Table 3).

### 3.2. Conversion Parameters and Substrate Mass Reduction

Feed had a significant effect on larval conversion efficiencies while no such effect was found significant in the case of density or the interaction between feed and density. Feed significantly affected the protein conversion efficiency (F_5,36_ = 124.97, *p* < 0.0001) which was higher on the larvae fed with CH.FEED reaching 45.77%, followed by BSG + BY, SCG + BSG + BY, BSG and SCG + BSG and lastly SCG + BY which had the lowest conversion rate with 17.55% (Table 4). Bioconversion rate (BCR) was significantly different in every diet case (F_5,36_ = 352.91, *p* < 0.0001), with higher values recorded in CH.FEED, BSG and SCG + BSG enriched with BY. Substrate mass reduction was also affected by feed (F_5,36_ = 1048.70, *p* < 0.0001) and was high in chicken feed resulting a value of 67.38% of the total feed added, followed by BSG and BSG + BY with 44.8% and 45.21%, respectively, and no statistical difference. Lower values were recorded in sole SCG + BSG while remarkably lower was the reduction of SCG + BY reaching 18.48% (Table 4).

### 3.3. Body Composition

**Figure 2:**
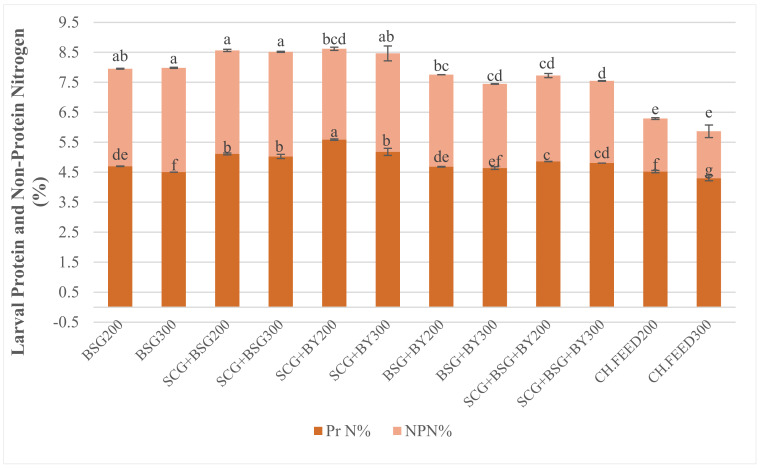
Effect of treatment, i.e., interaction between feed and density, on larval protein and non-protein nitrogen out of total nitrogen (% of DM), (mean ± standard error, *n* = 4). Mean values followed by different letter in the same level vary significantly (*p* < 0.05).

Nutrient composition of the BSF larvae fed on SCG, BSG, BY and their mixtures are shown in Table 5. Nutrient analysis of the larval bodies provided distinct indications that feed significantly influenced all tested body composition parameters (*p* < 0.0001). Interaction between feed and density was also significant in all cases except larval crude fat where, although feed (F_5,24_ = 63.86, *p* < 0.0001) and density (F_1,24_ = 28.95, *p* < 0.0001) exerted significant effects individually, no significant interaction was found. Total nitrogen ranged from 7.95 to 8.62% and was highest (F_5,24_ = 12.19, *p* < 0.0001) in treatments of sole BSG, SCG + BSG and SCG + BY at both densities (Table 5). Interestingly, the above treatments had the most increased non-protein nitrogen (F_5,24_ = 7.43, *p* = 0.0002) which reached up to 3.49%. The lowest NPN 1.77% and 1.56% respectively, was recorded on larvae fed CH.FEED at both densities. SCG + BY, BSG and CH.FEED diets at 200 larval density exhibited higher protein nitrogen (F_5,24_ = 9.46, *p* < 0.0001) and true protein (F_5,24_ = 10.69, *p* < 0.0001) compared to the corresponding treatments in 300 larval density. Overall, larvae fed sole feeds or mixtures with incorporated SCG, at both densities, showed significant higher protein nitrogen, with lower variations than values reported on total nitrogen, crude protein (F_5,24_ = 12.81, *p* < 0.0001) and true protein (Table 5; Figure 2). Concerning crude fat, factors feed and density exerted a significant impact on larvae reared on chicken feed and mixtures of BSG and SCG + BSG with BY resulting the highest percentages 26.71%, 25.21%, 25.28%, respectively, while on SCG + BSG accumulated the poorest ones reaching 15.97%. Larvae in density 300 gained 2.25% more fat than the ones reared on densities of 200, which is 23.85% in the former. Larvae fed on chicken feed had the highest percentage of Ash at 18.45% and 19.10%, while in the rest of the treatments Ash ranged from 7.22% to 8.13% (Table 5).

### 3.4. Correlation and Linear Regression

In order to identify any linear relationships between the various treatment parameters, correlation and linear regression analysis were conducted. Both positive and negative correlations were found between various nutritional properties of the feeds and mean larval dry mass (Table 6). Specifically, NDF and crude fat were negatively correlated with mean larval dry mass, in contrast to crude protein, NFC and ash which exhibited a positive correlation with mean larval dry mass. Linear regression analysis, showed that mean larval dry mass had a strict cause-effect relationship especially with crude protein and ash. Similarly, linear regression analysis for substrate mass reduction showed significant relationship with all ingredients, except NDF. Linear Regression equations on mean larval dry mass and conversion efficiencies were estimated:

Mean larval dry mass (y) = −0.028 + 0.265 (C. Protein) + 0.41 (ash)
PrCR (y) = 0.025 + 0.83 (CP) + 0.54 (Fat) − 0.44 (NFC) + 3.75 (Ash)
BCR (y) = −1.11 + 2.71 (Fat) + 1.93 (CP) + 1.28 (NDF) + 6.74 (Ash)
Substrate mass reduction (y) = 0.897 − 2.811 (C.P) + 2.178 (NFC) − 5.12 (Fat) − 8.80 (Ash)

## 4. Discussion

Black soldier fly larvae represent one of the most dominant contemporary trends in animal breeding for feedstock production and an important tool of managing and utilization of unfavorable food by-products [13,14,21]. As in all living organisms, biomass production is quantitatively and qualitatively related to diet composition and abiotic factors such as rearing density. In this study, we investigated the effect of two nutritionally low value, fibrous by-products (i.e., spent coffee grounds and brewer’s spent grains) individually or in combinations and after enrichment with brewer’s yeast on BSF larval survival, yield performance and body nutrient composition. Two rearing densities were used in order to investigate potential effect of density and interactions with the above tested diets.

Survival rate was high for all treatments exceeding 90.75%, except for SCG + BSG in the 300 larval density with 87.42% which is still high, indicating that the utilization of the low nutritionally value tested diets is not a forbidding factor, also demonstrating the remarkable resilience of the larvae in inhabiting materials such as sole spent coffee grounds. Survival rates on such diets are just as high as in other studies [35,36,37]. Development time until desirable stage completion was longer in the larvae fed SCG + BY while larvae fed solely with SCG were not able to achieve it. The first prevailing explanation is that these treatments were more readily dehydrated than the rest. The porosity or hydration properties of the diet can impact the conversion efficiencies. The capacity of the aforementioned diets (SCG, SCG + BY) could be characterized as swelling the water, BSG’s capacity as bind and the rest of them hold the water [38] and thus, the substrate dehydration may have stressed the larvae, resulting negative effects on their development. Secondly, low nutritional value of SCG may have led to larval diminished growth. Typically, insects require 8–10 essential amino acids to survive [39]. Early studies [40] have shown that coffee grounds have a low nitrogen content of roughly 2% (in our study SCG total nitrogen was 1.96 ± 0.17%), containing only half of the essential amino acids required for animal feed while the high NPN content (~46% of the total nitrogen) observed in SCG may partly explain its low biological effect observed in several animal feeding studies [7]. This suggests that hysteresis in valuable nutrients may have negatively impacted the BSF larval performance. In addition, the feeding ratio may have led to food limitation for the larvae as these diets had lower nutrient quality than the other diets. Development time for larvae fed with diets based on BSG is compliant with previous studies. Chia et al. [35] found that development of larvae fed with brewery wastes in various combinations lasted totally 17–21 days, while in the present study total larval development lasted 13–14 days. Meneguz et al. [36] reared BSF larvae in brewery wastes *ad libidum* and reported that development lasted 8 days in trial, while in lower nutritional value winery by-products the duration was 22.2 days.

Statistical analysis showed that the mean larval and pre-pupal wet and larval dry mass were affected by the kind of feed rather than density or the interaction of the two factors. The chicken feed produced the largest larvae but their development lasted 3 days longer than the next feed, i.e., BSG + BY, followed by SCG + BSG + BY. It was evident that SCG had a negative effect on performance parameters as indicated by the significant declined larval mass whenever SCG was a part of a diet (Figure 1). Whether this is due to some specific growth-inhibitory substance of this substrate is under question. Fischer et al. tested the performance of BSF larvae on sole and blends of spent coffee grounds and donuts where they proved that the former developed the smallest [41]. SCG contains various nitrogen-containing substances such as caffeine, trigonelline, free amines and amino acids [42]. It is possible that, even though the caffeine content of SCG is reported to be low (0.734 to 41.3 μg/mg of SCG extracts) it may have an impact on BSF larval development [7]. Laranja et al. [43] indicated that caffeine and used coffee grounds completely blocked the development of *Aedes aegypti* in the early stages, in treatments with concentrations of 1.0 mg/mL and 50 mg/mL, respectively, whereas observations of the time of larva and adult onset suggested that developmental time was also delayed in treatments with both substances. In another experiment, Nikitin et al. [44] observed a significant reduction in the life span of male *Drosophila melanogaster* flies reared on food containing 2.5 mg/mL and 1.25 mg/mL of caffeine.

Overall, our study demonstrated that the nutrients that had a cause-effect relationship with mean larval dry mass were substrate crude protein and ash. Chicken feed, being a conventional feed, had comparatively low CP but probably a more balanced amino acid profile and by far the highest ash content. In a similar study by Lalander et al. [45], crude protein and ash exerted a pivotal role on larval performance in eight urban organic waste fractions, demonstrating that volatile solids and crude protein of the diets had the greatest impact on biomass conversion rate and larval development time.

Feed had a significant effect in all conversion parameters. BSF larvae fed on chicken feed had the higher PrCR and BCR which means that larvae utilized these diets in a more efficient way. It was evident that PrCR of BSG and SCG + BSG feeds was significantly increased when they were enhanced with BY although they could not reach chicken feed levels. Linear regression, PrCR (y) = 0.025 + 0.83 (CP) + 0.54 (Fat) − 0.44 (NFC) + 3.75 (Ash), showed that PrCR is related with all diet components, except for NDF. Even though this was expected as NDF does not provide significant nutritional elements to the larvae, regression analysis indicated that other factors than protein content influence this parameter. On the contrary, BCR, which is related to larval biomass, showed significant differences in all feed treatments. According to regression analysis BCR (y) = 1.11 + 2.71 (Fat) + 1.93 (CP) + 1.28 (NDF) + 6.74 (Ash), all components, except NFC, are related to the Bioconversion rate. SCG + BY and sole SCG + BSG bioconversion values were between 6.25–8.22 and thus comparable to values assessed in previous experiments that tested used fruits and vegetables, poultry and dairy manure [45,46]. For chicken feed, BSG + BY and SCG + BSG + BY bioconversion values were between 14.16–23.28, similar to diets comprising of human faeces, mill by-products, canteen wastes and poultry feed [47,48]. Substrate mass reduction was lower for all of the treatments in comparison to the chicken feed, with SCG + BY having the poorest reduction performance followed by sole SCG + BSG. This could be due to high NDF% content, i.e., cellulose, hemicellulose and lignin, which is hardly digested from the larvae. Previous studies have proven that lignin in combination with low crude protein and non-fiber carbohydrates exert a significantly negative effect on larval growth, [49]. However, lignocellulosic percentage was similar for the sole BSG diet, which resulted in a 45.21% mass reduction, which was not significantly different to BSG + BY. Mass reduction is connected directly with larval performance, so factors affecting larval performance analyzed above may also account for the observed poor bioconversion percentage. Interestingly, linear regression, Substrate mass reduction (y) = 0.897 − 2.811 (CP) + 2.178 (NFC) − 5.12 (Fat) − 8.80 (Ash), showed that all ingredients of the feed, except NDF%, exerted a cause-effect relationship. As NDF parameters do not contribute to larval biomass increase and nutrient assimilation, reduction of the diet increases as the rest of the nutrients are increasing. A substrate with high NDF content, especially lignin, could be expected to be broken down less efficiently by BSFL composting, thus resulting in nutritionally poorer and residue richer substrate.

Even though feed as factor exerted significant effect on mean larval mass, nutrient analysis of the larvae revealed interaction between feed and larval density on BSF larval nitrogen levels (Table 5). Diets who resulted the lowest mean larval wet and dry mass, exhibited the highest total nitrogen levels and crude protein. Differences among treatments were significant but not great, except in the case of chicken feed. Total nitrogen and crude protein for larvae fed with chicken feed, BSG + BY and SCG + BSG + BY in the 1.45 density differed significantly and exhibited higher values than the corresponding larvae in the 2.17 density, i.e., 27.92–36.92%. Higher larval CP content was recorded on sole BSG, SCG + BSG and SCG + BY regardless the density ranging between 37.89% and 41.03% with no statistical differences. In general, differences between densities inside the same diet were small, while diets which contained spent coffee grounds exerted higher CP, much more than that of larvae fed with chicken feed. Larval crude fat may contribute to these observed differences.

Both density and feed affected larval fat accumulation, with larvae fed with chicken feed, BSG + BY and SCG + BSG + BY reached the highest fat percentages in contrast, with CP that was the lowest, compared to the other treatments. This could mean that a trade-off between protein levels and stored fat exists or that a minimum protein threshold level has to be reached rapidly in the pre-pupal stage before fat storage can take place [50]. Insects are able to compensate for an unbalanced diet through both behavioral and physiological mechanisms to obtain an optimal intake of energy and nutrients [51]. Fat is of great importance for BSF since it is utilized for pre-pupa wandering and adult reproduction [52]. Barragan-Fonseca et al. [53] demonstrated that heavier BSF larvae, as well as larvae reared on high dietary protein and NFC resulted in higher crude fat content. It has also been shown that larval crude protein content was higher in larvae fed on the diet with the lowest crude protein content [19,54]. These findings are in line with our results since larvae fed on sole BSG, SCG + BSG as well as SCG + BY had higher larval crude protein, while the diets BSG + BY, SCG + BSG + BY with higher dietary protein resulted in larvae with increased crude fat content. However, chicken feed which had comparatively low dietary CP produced larvae with the lowest larval CP but it contained by far the highest NFC and ash percentage which presumably means that larvae ceased the protein assimilation but continued to store the indispensable for them fat.

Nevertheless, not only does dietary crude protein exert strong effects on growth, but also different amino acid and NFC profile of the feeds alter the BSF larval performance [15]. Most insects acquire their amino acids from proteins, rather than from the fairly limited pool of free amino acids [39]. Due to protein’s overestimation from previous studies, we also measured non-protein nitrogen and consequently calculated the true protein [15,55]. It was common practice to calculate crude protein as total nitrogen multiplied by the standard Kp factor 6.25, which means that in 100 g of protein 16 g of nitrogen are contained. The presence of non-protein nitrogen (NPN) in insects, for example, chitin, nucleic acids, phospholipids and excretion products (e.g., ammonia) in the intestinal tract, could lead to an overestimation of the protein content. In the drive of this knowledge, Janssen et al. [30] proposed a Kp value of 4.76 to be adopted for determining of protein content of BSF larvae to avoid overestimation, which means that 100 g insect protein contain 21 g of nitrogen. We observed that the previous claim, according to interactions between dietary and larval crude protein, is also consistent with true protein results. Our study showed that SCG + BSG and SCG + BY which had the lowest dietary protein among treatments result larvae with higher true protein 23.94–26.61%. Only larvae which were fed with the control diet sustained low NPN levels, while the rest of the treatments had small significant differences with NPN ranging from 2.74 to 3.49.

## 5. Conclusions

BSF larvae were able to efficiently consume and utilize all given fibrous diets which were proven to be suitable for the insect’s development, except in the case of sole spent coffee grounds. Overall, feeding on BSG + BY and SCG + BSG + BY resulted enhanced larval performance, conversion parameters and body composition close to the control feed. Density did not have a significant effect on larval properties except for crude fat. The interaction between feed and density strongly affected nitrogen and protein levels. Furthermore, we found that larvae produced a true protein range between 20.48% to 26.61%, much lower than values reported for crude protein content in the literature, ranging from 37% to 63% [54,56]. We propose that spent coffee grounds should participate in the diets in lower amounts so as to minimize the adverse consequences and simultaneously exploit this affluent by-product. In further studies, amino acid and NPN profile of substrates and larvae has to be determined in order to explain the potential alterations. Additional experiments may be performed to show if caffeine has a negative impact on larval growth.

## Figures and Tables

**Table 1:** Chemical properties of spent coffee ground, brewer’s spent grain, brewer’s yeast with their combinations used in the current study (mean ± standard error; *n* = 3).

Substrate	C. Protein%	C. Fat%	NDF%	ADF%	Ash%	NFC%
**SCG**	10.60 ± 0.93	13.43 ± 0.01	52.12 ± 0.19	29.16 ± 0.06	1.22 ± 0.01	22.74 ± 0.66
**BSG**	18.72 ± 0.29	4.69 ± 0.06	50.05 ± 0.27	18.60 ± 0.23	3.80 ± 0.01	22.89 ± 0.04
**SCG + BSG**	14.62 ± 0.10	9.54 ± 0.03	52.09 ± 0.42	24.48 ± 0.28	2.35 ± 0.18	21.03 ± 0.10
**SCG + BY**	15.36 ± 0.40	10.97 ± 0.09	44.45 ± 0.95	24.48 ± 0.52	2.64 ± 0.60	26.86 ± 2.11
**BSG + BY**	21.12 ± 0.04	3.91 ± 0.05	38.24 ± 0.84	14.45 ± 0.36	4.59 ± 0.01	31.30 ± 0.16
**SCG + BSG + BY**	18.52 ± 0.03	7.66 ± 0.17	44.63 ± 1.71	20.87 ± 0.84	3.66 ± 0.02	26.79 ± 1.83
**CH.FEED**	14.96 ± 0.16	3.01 ± 0.02	13.50 ± 0.16	4.50 ± 0.07	11.69 ± 0.35	57.00 ± 0.25

**Table 2:** Development time, survival rate, dry matter (DM%) and larval yield (g in DM) of larvae reared on spent coffee ground (SCG), brewer’s spent grains (BSG), brewer’s yeast (BY) and their mixtures (mean ± standard error *n* = 4). Mean values followed by different letters in the same column vary significantly (*p* < 0.05).

Treatment	Development Time (Days)	Survival Rate (%)	Larval DM (%)	Larval Yield (g in DM)
**SCG 200**	35	96.37 ± 0.37 ^a,b^	24.68 ± 0.40 ^f^	0.61 ± 0.05 ^i^
**SCG 300**	35	97.66 ± 0.59 ^a,b^	25.52 ± 0.41 ^f^	0.77 ± 0.02 ^i^
**BSG 200**	9	99.38 ± 0.38 ^a^	30.29 ± 0.06 ^b,c^	6.02 ± 0.16 ^e,f^
**BSG 300**	9	99.33 ± 0.41 ^a^	29.90 ± 0.22 ^b,c^	8.53 ± 0.13 ^c,d^
**SCG + BSG 200**	12	99.38 ± 0.38 ^a^	25.99 ± 0.11 ^e,f^	4.19 ± 0.02 ^g^
**SCG + BSG 300**	12	87.42 ± 6.79 ^b^	26.17 ± 0.53 ^e,f^	6.10 ± 0.03 ^e,f^
**SCG + BY 200**	17	90.75 ± 3.86 ^a,b^	25.14 ± 0.49 ^f^	2.99 ± 0.46 ^h^
**SCG + BY 300**	17	94.25 ± 2.39 ^a,b^	25.98 ± 0.40 ^e,f^	5.06 ± 0.60 ^f,g^
**BSG + BY 200**	8	98.25 ± 1.75 ^a,b^	33.07 ± 0.35 ^a^	8.40 ± 0.06 ^c,d^
**BSG + BY 300**	8	99.00 ± 0.58 ^a^	31.20 ± 0.55 ^a,b^	12.72 ± 0.04 ^b^
**SCG + BSG + BY 200**	12	99.00 ± 0.20 ^a^	29.01 ± 0.21 ^c,d^	6.93 ± 0.04 ^d,e^
**SCG + BSG + BY 300**	12	98.75 ± 0.75 ^a^	27.63 ± 0.16 ^d,e^	10.53 ± 0.47 ^b,c^
**CH. FEED 200**	11	99,88 ± 0,13 ^a^	32,83 ± 0,19 ^a^	11.32 ± 0.15 ^b^
**CH. FEED 300**	11	100 ^a^	31,22 ± 0,64 ^a,b^	16.78 ± 0.34 ^a^

**Table 3:** Mean larval and pre-pupal wet and larval dry mass of the BSF larvae reared on SCG, BSG, BY and their mixtures (mean ± standard error, *n* = 4). Mean values followed by different letter in the same column vary significantly (*p* < 0.05). NM: Not measured.

Feed	Mean Larval Wet Mass (g)	Mean Larval Dry Mass (g)	Mean Pre-Pupal Wet Mass (g)
**SCG**	0.0115 ± 0.0007 ^f^	0.0029 ± 0.00017 ^f^	NM
**BSG**	0.0989 ± 0.0019 ^c^	0.0294 ± 0.00058 ^c^	0.0871 ± 0.0012 ^c^
**SCG + BSG**	0.0864 ± 0.003 ^d^	0.0223 ± 0.001 ^d^	0.0744 ± 0.0047 ^c,d^
**SCG + BY**	0.0659 ± 0.0042 ^e^	0.017 ± 0.0012 ^e^	0.0733 ± 0.0041 ^d^
**BSG + BY**	0.1349 ± 0.0022 ^b^	0.0428 ± 0.00031 ^b^	0.1186 ± 0.0017 ^b^
**SCG + BSG + BY**	0.1248 ± 0.0027 ^b^	0.0352 ± 0.00065 ^c^	0.1202 ± 0.0035 ^b^
**CH.FEED**	0.1774 ± 0.002 ^a^	0.0563 ± 0.00066 ^a^	0.1601 ± 0.0032 ^a^

**Table 4:** Protein conversion rate, bioconversion rate and mass reduction after feeding with tested diets (mean ± standard error *n* = 4). Mean values followed by different letter in the same column vary significantly (*p* < 0.05). NM: Not measured.

Feed	PrCR	BCR	Substrate Mass Reduction
**SCG**	NM	NM	NM
**BSG**	24.75 ± 0.44 ^c^	11.83 ± 0.22 ^d^	45.21 ± 0.38 ^b^
**SCG + BSG**	23.91 ± 0.18 ^c^	8.22 ± 0.06 ^e^	26.64 ± 0.35 ^d^
**SCG + BY**	17.55 ± 1.60 ^d^	6.25 ± 0.61 ^f^	18.48 ± 0.81 ^e^
**BSG + BY**	30.15 ± 0.21 ^b^	17.22 ± 0.07 ^b^	44.80 ± 0.46 ^b^
**SCG + BSG + BY**	28.54 ± 0.60 ^b^	14.16 ± 0.31 ^c^	33.08 ± 0.45 ^c^
**CH.FEED**	45.77 ± 0.64 ^a^	23.28 ± 0.16 ^a^	67.38 ± 0.31 ^a^

**Table 5:** Nutrient composition of the BSF larvae fed on SCG, BSG, BY and their mixtures (mean ± standard error *n* = 3). Mean values followed by different letter in the same column vary significantly (*p* < 0.05). NM: Not measured.

Treatment	Total N	Protein N	NPN	C. Protein	True Protein	Ash
**SCG 200**	NM	NM	NM	NM	NM	NM
**SCG 300**	NM	NM	NM	NM	NM	NM
**BSG 200**	7.95 ± 0.01 ^b^	4.70 ± 0.01 ^d,e^	3.25 ± 0.02 ^a,b^	37.86 ± 0.03 ^b^	22.37 ± 0.05 ^d,e^	7.34
**BSG 300**	7.98 ± 0.02 ^b^	4.51 ± 0.001 ^f^	3.48 ± 0.02 ^a^	38.00 ± 0.09 ^b^	21.45 ± 0.01 ^f^	7.61
**SCG + BSG 200**	8.56 ± 0.004 ^a^	5.11 ± 0.03 ^b^	3.45 ± 0.04 ^a^	40.76 ± 0.02 ^a^	24.33 ± 0.15 ^b^	7.82
**SCG + BSG 300**	8.52 ± 0.05 ^a^	5.03 ± 0.07 ^b^	3.49 ± 0.02 ^a^	40.54 ± 0.25 ^a^	23.94 ± 0.34 ^b^	7.67
**SCG + BY 200**	8.62 ± 0.03 ^a^	5.59 ± 0.03 ^a^	3.03 ± 0.05 ^b,c,d^	41.03 ± 0.14 ^a^	26.61 ± 0.12 ^a^	7.35
**SCG + BY 300**	8.47 ± 0.13 ^a^	5.18 ± 0,.2 ^b^	3.29 ± 0.25 ^a,b^	40.30 ± 0.62 ^a^	24.66 ± 0.57 ^b^	7.32
**BSG + BY 200**	7.76 ± 0.01 ^c^	4.69 ± 0.01 ^d,e^	3.07 ± 0.001 ^b,c^	36.92 ± 0.07 ^c^	22.30 ± 0.06 ^d,e^	8.06
**BSG + BY 300**	7.45 ± 0.03 ^d^	4.64 ± 0.04 ^e,f^	2.81 ± 0.01 ^c,d^	35.45 ± 0.15 ^d^	22.08 ± 0.21 ^e,f^	8.13
**SCG + BSG + BY 200**	7.73 ± 0.06 ^c^	4.86 ± 0.004 ^c^	2.87 ± 0.07 ^c,d^	36.78 ± 0.31 ^c^	23.13 ± 0.02 ^c^	7.22
**SCG + BSG + BY 300**	7.55 ± 0.01 ^d^	4.81 ± 0.001 ^c,d^	2.74 ± 0.01 ^d^	35.93 ± 0.05 ^d^	22.89 ± 0.001 ^c,d^	7.75
**CH.FEED 200**	6.29 ± 0.01 ^e^	4.52 ± 0.04 ^f^	1.77 ± 0.03 ^e^	29.95 ± 0.04 ^e^	21.53 ± 0.18 ^f^	19.10
**CH.FEED 300**	5.87 ± 0.12 ^f^	4.30 ± 0.08 ^g^	1.56 ± 0.21 ^e^	27.92 ± 0.59 ^f^	20.48 ± 0.40 ^g^	18.45

**Table 6:** Pearson correlation coefficients between nutritional parameters and mean larval dry mass analysis.

Correlation Coefficients	C. Protein%	NDF%	Larval Mean Dry Mass	Non-Fiber Carbs%	Fat%	Ash%
C.Protein%	1	NS	0.6024 ***	NS	−0.7058 ***	NS
NDF%	-	1	−0.8026 ***	−0.9956 ***	0.6449 ***	−0.9666 ***
Larval Mean Dry Mass	-	-	1	0.7749 ***	−0.9255 ***	0.8688 ***
NF C%	-	-	-	1	−0.6177 ***	0.9615 ***
Fat%	-	-	-	-	1	−0.7617 ***
Ash%	-	-	-	-	-	1

*** indicate significant correlations at a 0.01 level.

## Data Availability

The data presented in this study are available on request from the corresponding author. The data are not publicly available due to also forming part of an ongoing study.
